# Analysis of mutant and total huntingtin expression in Huntington’s disease murine models

**DOI:** 10.1038/s41598-020-78790-5

**Published:** 2020-12-17

**Authors:** Valentina Fodale, Roberta Pintauro, Manuel Daldin, Roberta Altobelli, Maria Carolina Spiezia, Monica Bisbocci, Douglas Macdonald, Alberto Bresciani

**Affiliations:** 1 Department of Translational Biology, IRBM S.p.A., via Pontina Km 30, 600 Pomezia, Rome, Italy; 2 CHDI Management/CHDI Foundation, Suite 700, 6080 Centre Drive, Los Angeles, CA USA

**Keywords:** Huntington's disease, Motor neuron disease, Movement disorders, Neurodegeneration, Immunological techniques, Mouse, Rat, Biomarkers, Pharmacodynamics

## Abstract

Huntington’s disease (HD) is a monogenetic neurodegenerative disorder that is caused by the expansion of a polyglutamine region within the huntingtin (HTT) protein, but there is still an incomplete understanding of the molecular mechanisms that drive pathology. Expression of the mutant form of HTT is a key aspect of diseased tissues, and the most promising therapeutic approaches aim to lower expanded HTT levels. Consequently, the investigation of HTT expression in time and in multiple tissues, with assays that accurately quantify expanded and non-expanded HTT, are required to delineate HTT homeostasis and to best design and interpret pharmacodynamic readouts for HTT lowering therapeutics. Here we evaluate mutant polyglutamine-expanded (mHTT) and polyglutamine-independent HTT specific immunoassays for validation in human HD and control fibroblasts and use to elucidate the CSF/brain and peripheral tissue expression of HTT in preclinical HD models.

## Introduction

Understanding the pathophysiological mechanisms of monogenic diseases such as Huntington’s disease (HD) requires investigation of steady state levels of the mutated protein in various tissues, and in the presence of pharmacological and genetic modulators. Immunoassays using specific antibodies are the most common way to quantify such proteins, and validation of these immunoassays is essential for meaningful data interpretation and understanding pathobiology.

HD is a neurodegenerative disorder caused by an expansion of a CAG triplet repeat in the *huntingtin* (*HTT*) gene^1^, which results in the production of a mutant HTT protein (mHTT) with an expanded polyglutamine (poly-Q) domain in exon 1. Expansions greater than 35 glutamines lead to neuronal loss associated with motor and behavioural disorders^2^. HTT biology has been extensively studied^3^ but the precise function of HTT remains to be clarified. Reducing the amount of mHTT expressed is one of the most promising therapeutic interventions, with several modalities in clinical trials and late stage preclinical development^4–6^. Other therapeutic approaches include HTT post-translational modification associated clearance and function^7–11^. The ability to design pharmacodynamic readouts together with the proper measure of mHTT/HTT levels is central to interpreting efficacy results with these potential therapeutics.

To date, the CSF mHTT concentration has been used as a pharmacodynamic readout for HTT lowering interventions in both clinical trials^6^ and pre-clinical studies^12,13^, using a polyQ-dependent relative-quantitative assay employing the 2B7 and MW1 monoclonal antibodies^6,14^. Despite this fact, published reports describing quantitative brain and CSF levels of mHTT in preclinical species are limited^12,15^ and completely missing for total (poly-Q independent) HTT. A comparison of total HTT levels with mutant HTT will offer a better picture of the pharmacological effects of investigational drugs. In addition, the understanding of total HTT protein expression in various tissues is important to understand relative differences in the expression of mutant HTT levels across tissues and time.

Several antibodies and technologies such as TR-FRET^16^, Mesoscale Discovery (MSD)^17,18^, microbead-based immunoprecipitation followed by flow cytometry (IP-FCM)^12^, and single molecule counting (SMC)^19^ have all been used to quantify mHTT/HTT, with SMC becoming the assay of choice due to its ultra-sensitivity which allows for mHTT measurement in human cerebrospinal fluid^20^. Here we initially set out to systematically investigate the SMC platform for mHTT/HTT detection assays that use N-terminal directed antibodies with the aim of generating guidelines on their use and interpretation. Subsequently we used the best-performing assays to demonstrate that CSF and brain mHTT levels in a BACHD rat model^21^ correlate well throughout its lifespan. Finally, we determined mHTT/HTT levels in the zQ175 mouse model of HD^22^ to investigate variance of HTT expression in different tissues and during aging.

## Results

### Antibody and standard protein identity and quality

**Figure 1:**
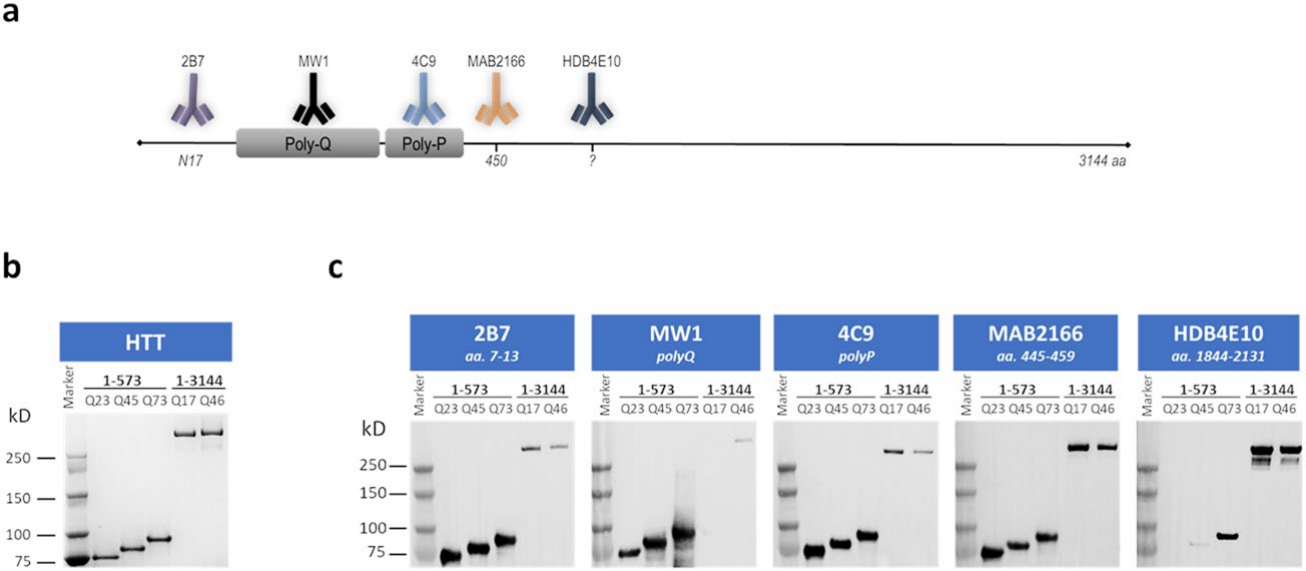
Reagent qualification. (**a**) Schematic of the HTT protein and the location of the epitope of each antibody used in the present work (the protein length is not properly scaled). (**b**) Coomassie staining of the five standard protein used in the present work (left to right for each panel: N573 Q22, N573 Q45, N573 Q72, FL Q17, FL Q46). (**c**) Western blots of the five standard proteins (left to right for each panel: N573 Q22, N573 Q45, N573 Q72, FL Q17, FL Q46) stained with the five antibodies used in the present work. Figure S-1 shown the full length gels and the high exposure full length Western blots.

In the present work, a panel for N-terminal HTT directed antibodies were used to investigate HTT detection by SMC assays. For this reason we excluded the D7F7 antibody from this attempt. Nonetheless, in consideration of the relevance of this antibody, we are working on a separated report dedicated to full length HTT/mHTT detection assays. After review of the wide variety of commercial and non-commercial antibodies against this HTT region, we selected five to investigate (Fig. 1a). The 2B7 antibody^16,23^ was selected because, together with the MW1^24^, it is currently used for mHTT detection in mHTT lowering clinical studies^4,19,20^. The MW1 antibody was selected for the above reason and for being the most used poly-Q directed antibody^18,19,25^. In addition to the poly-Q domain, there is a proline rich domain (PRD) within the HTT exon 1 which is recognized by the 4C9 antibody^16^ that was included. In fact, the 2B7-4C9 pair is reported to be the most N-terminal poly-Q independent HTT detection assay^18^. Further down the HTT sequence, the MAB2166 antibody^26^ was used as it is widely recognized to be one of the most HTT specific antibodies by western blots^18^. Finally the HDB4E10 antibody^27^, was selected because it has been used for mHTT detection in preclinical CSF samples and potentially clinical samples^12^. We then used five variants of recombinant HTT proteins as standards for quantification. Two protein lengths were chosen to investigate size-dependant detection: 1-3144 amino acid full length HTT (FL) and a 1-573 amino acid N-terminal fragment (N573). In addition, to assess the poly-Q dependency of HTT detection two FL human proteins with poly-Q expansion repeats of Q17 and Q46 were used and three N573 fragment HTT proteins with a Q23, Q45, and Q73 were used. The identity and quality of these proteins were investigated by SDS-PAGE (Fig. 1b). They were all confirmed at the expected molecular weight based on the protein size plus the poly-Q expansion, with no evident contaminants. The quality of the antibodies was assessed by Western blot (WB) on the five reference proteins (Fig. 1c and S-1). All tested antibodies were able to recognize all reference proteins. Beyond some minor differences that are likely attributable to variable loading, the MW1 antibody, as expected was confirmed to recognize the higher poly-Q repeat proteins with greater efficiency. Surprisingly, the HDB4E10, which was reported to bind between position 1844 and 2131, was able to recognize the N573 proteins in a poly-Q dependent manner (Fig. 1c). As a consequence it was hypothesized that this antibody was accidentally raised against one or more HTT epitopes different than the one claimed by Wilkinson et al.^27^.

### Performance of the standard HTT proteins (selectivity)

**Figure 2:**
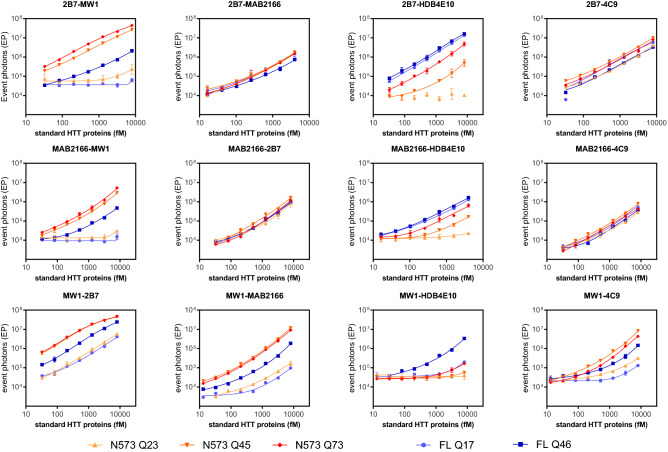
Performance of the standard curve detection by all antibody pairs. The five standard HTT proteins (N573 Q22, N573 Q45, N573 Q72, FL Q17, FL Q46) were serially diluted from 8 pM and assayed via SMC using the antibody pair reported on the title of each chart; the first mentioned antibody was used as capture whereas the second was used as detection. Each experimental point is reported as the average and standard deviations of a triplicate.

The SMC assay is an immunoassay where one antibody is immobilized on magnetic particles (MPs) for analyte capture while the detection antibody is conjugated with a fluorophore for quantification. Among the selected antibodies, the HDB4E10 was excluded from testing as capture antibody because of the unexpected HTT recognition pattern with respect to what is claimed in literature^27^. However it was used as a detection antibody to validate the results reported by Southwell et al.^12^ Another antibody which was excluded from being used as capture was 4C9; in fact the initial attempts to conjugate it with magnetic particles very often resulted in hard to re-suspend clumps. In conclusion, the 2B7, MW1, and MAB2166 were tested as capture antibodies in combination with the other five antibodies as detection, Lastly, homo-pairing of capture and detection antibodies was excluded. Figure 2 shows a matrix of graphs with the three capture antibodies by row and the detection antibodies by columns. In each graph, the SMC signal generated by serial dilutions of the five reference HTT proteins is plotted. As expected by the fact that the MW1 and the HDB4E10 recognizes HTT in a poly-Q dependent manner, all assays where at least one of these antibodies is present (regardless as capture or detection) resulted in a poly-Q dependant signal. In addition, the N573 HTT and the FL HTT proteins with similar poly-Qs (45 and 46 respectively), were differently detected when the MW1 or the HDB4E10 were present suggesting that the size of the HTT protein influences the detection. Further to this, while MW1 preferentially detects the N573 proteins (as previously described)^19^, the HDB4E10 antibody display a higher signal for the FL proteins with the 2B7-HDB4E10 and the MAB2166-HDB4E10 being poly-Q independent for only the FL proteins. Finally, when MW1 containing reverse pairs are considered (i.e. 2B7-MW1 vs MW1-2B7 or MAB2166-MW1 vs MW1-MAB2166) the assays with the MW1 as detection were more selective for mHTT with respect to HTT. On the other hand, the 2B7-4C9, the 2B7-MAB2166, the MAB2166-2B7, and the MAB2166-4C9 assays resulted in a poly-Q and protein length independent detection of all reference proteins. These aspects are relevant to data analysis and interpretation.

### Specificity for mHTT, dilution linearity and spike recovery in wild type and BACHD rat brain homogenates

Following the assessment of reference HTT protein detection, the selected panel of assays were tested on wild type (wt) and BACHD rat brain homogenates. The choice of BACHD rats was based on the relative ease of collection and amount of CSF versus mouse models of HD.

**Figure 3:**
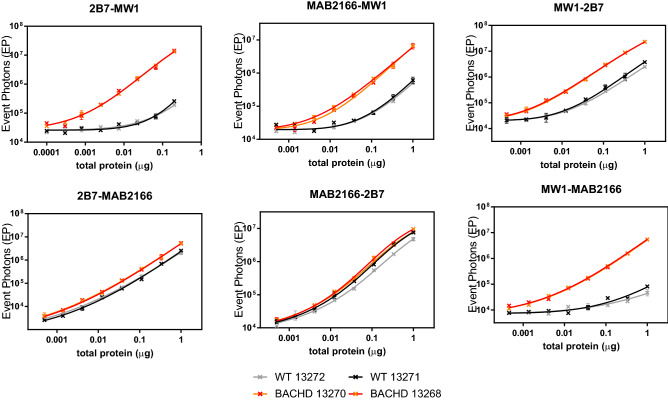
mHTT specificity of the detection in wild type and BACHD rat brain homogenates. Two wild type rats (13272 and 13271 grey and black respectively) and two BACHD rats (13270 and 13266 red and orange respectively) brain homogenates were diluted starting from 1.3 µg/ml total protein for the 2B7-MW1 assay and from 6.7 µg/ml total protein for all other antibody pairs. Each dilution curve was assayed via SMC using the antibody pair reported on the title of each chart. Each experimental point is reported as the average and standard deviations of a triplicate. Results for assays containing the HDB4E10 and 4C9 antibodies are reported in Fig. S-3.

The BACHD rat expresses a full length human HTT transgene on an artificial bacterial chromosome and two copies of the wildtype rat HTT^27^. The poly-Q expansion of the transgene is expected to be around 97 glutamines. A mixed CAG/CAA coding sequence for the poly-Q domain abolish genetic instability in repeated DNA regions^21^. Endogenous rat HTT is expected to have 8 glutamines and a poly-Pro region with low similarity to the human sequence^28^. The specificity of the detection of the human mHTT transgene, with respect to endogenous rat, was assessed for each antibody pair by assaying serial dilutions of two BACHD rat and two wt brain homogenates (Fig. 3 and Fig. S-3). As anticipated, all assays using the 4C9 antibody as detection (i.e. directed against the human poly-Pro) displayed very low sensitivity. In addition, the 2B7-4C9 and the MAB2166-4C9 assays, whose detection was poly-Q independent on reference proteins, appears poly-Q dependent on these tissues. Instead, the 2B7-MAB2166, the MAB2166-2B7 and, to a less extent, the MAB2166-HDB4E10 assays confirmed as poly-Q independent assays. The detection of all other combinations resulted poly-Q dependent with the 2B7-MW1 and the MW1-MAB2166 assays being the most optimal mHTT specific antibody pairs.

**Figure 4:**
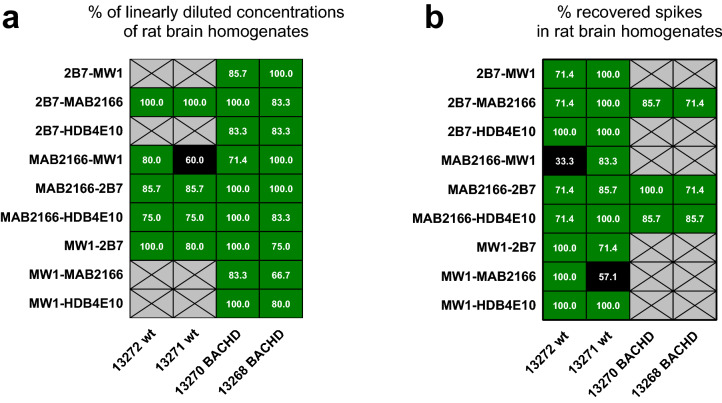
Dilution linearity and spike recovery in wild type and BACHD rat brain homogenates. Grey crossed cells represent not tested antibody pairs, black cells are antibody pairs that did not meet the 67% acceptance criteria (see text), green cells show antibody pairs with achieved dilution linearity and/or spike recovery. (**a**) Two wild type rats(13272 and 13271) and two BACHD rats (13270 and 13266) brain homogenates were diluted starting 1.3 µg/ml of total protein for the 2B7-MW1 assay and from 6.7 µg/ml of total protein for all other antibody pairs for 7 dilution points. Each dilution curve was assayed via SMC using the antibody pair reported on the x-axis. The percentage of the dilution points within ± 20% RE% is reported in each cell of the heat-map. The acceptance threshold is 66.7%; all passed conditions are coloured in green. All data for each matrix and assay is reported in the supplementary figure S-4 along with an example of the dilution linearity calculations. The Dilution linearity of wild type homogenates for the 2B7-MW1, 2B7-HDB4E10, MW1-MAB2166, and MW1-HDB4E10 are not reported as most dilution points were below the LLoQ for these assays. (**b**) The same four rat brain samples were spiked with a serial dilution of the recombinant FL HTT Q46 and assayed via SMC using the antibody pair reported on the x-axis. The percentage of the recovered spikes, within one matrix, are in each cell of the heat-map. The acceptance threshold is 66.7%; all passed conditions are coloured in green. All data for each matrix and assays are reported in the supplementary figure S-5. The spike recovery test was not run in BACHD rat homogenates with assays including the MW1 antibody as the signal of the endogenous analyte was too high.

A valid immunoassay must be compliant with the dilution linearity and spike recovery tests. Dilution linearity is achieved when the signal obtained at each sample dilution, multiplied by the respective dilution factor, falls within ± 20% relative error percentage (RE%) for at least 67% of the tested dilutions (4-6-20% rule)^29–31^. The assays with the 4C9 as detection antibody were excluded from the dilution linearity (and spike recovery) test due to low sensitivity. The poly-Q dependent assays (i.e. those with the MW1 and the HDB4E10 antibodies) passed the dilution linearity test for BACHD rat homogenates while wt homogenates were linearly diluted only for samples well above the lower limit of quantification (LLoQ) (S-4 and Fig. 4a). Instead, the 2B7-MAB2166 and the MAB2166-2B7 assays, which are poly-Q independent, resulted positive in this test both for BACHD rat and wt homogenates.

Spike recovery provides an indication of the matrix interference on analyte detection. In this case at least 67% of the spiked dilutions must be recovered within ± 20% of the nominal spike concentration. Spike recovery is carried out by measuring the ability of an assay to recover known concentrations of the reference analyte when spiked in a matrix which possibly doesn’t contain the analyte. As a consequence, wt rat brain homogenates were used as matrix for mHTT specific assays with FL Q46 spiked as reference analyte. All of these assays passed this test with at least one of the two wt brain homogenate (S-5 and Fig. 4b). With regard to the poly-Q independent assays (2B7-MAB2166, MAB2166-2B7and MAB2166-HDB4E10) a spike recovery test was run in presence of a mid-low homogenate concentration of both wt and BACHD origin in order to minimize the endogenous analyte influence. These assays passed the spike recovery test.

### Specificity for mHTT, dilution linearity, and spike recovery in primary human HD and control fibroblasts

**Figure 5:**
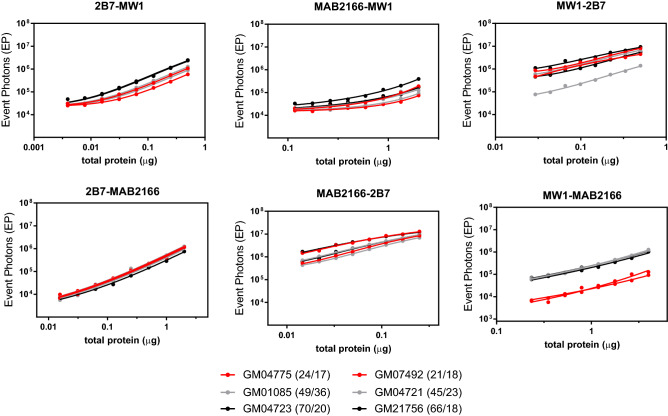
mHTT specificity of the detection in normal, HD, and JHD human fibroblast lysates. Two control GM04775 (24/17) and GM07492 (21/18), two HD GM04721 (49/36) and GM01085 (45/23) and two JHD GM04723 (70/20) and GM21756 (66/18) fibroblast cell lysates were diluted and assayed via SMC using the antibody pair reported on the title of each chart. Due to the large difference in sensitivity among the various antibody pair, the x-axis scale is not directly comparable for all charts. Each experimental point is reported as the average and standard deviations of a triplicate. Results for assays containing the HDB4E10 and 4C9 antibodies are reported in Fig. S-6.

Although extremely useful for pre-clinical studies, the detection of mHTT/HTT in animal model tissues may have some limitations. In fact, in animal models the poly-Q expansions required to generate HD phenotypes are typically much higher (> 100 Q) than the pathologic human HD repeats (> 35 Q). In addition, mHTT is introduced via multiple copies of an artificial allele^32,33^ or by knocking-in the human exon 1 into the animal *Hdh* locus^34^. Finally the animal background poly-Qs are typically lower (< 10) than ones in human normal HTT (~ 20)^28^. In order to investigate the performance of the present assays in a human context, two high expansion HD samples (juvenile HD, JHD), two average expansion HD samples (HD), and two unaffected control (normal) primary human fibroblasts were used (S-2, S-6 and Fig. 5). Similarly, to what was observed in rat derived samples the assays with the 4C9 antibody as detection resulted in poor sensitivity. In addition, the MAB2166-MW1 assay was also not found to be sensitive. The 2B7-MAB2166, the 2B7-HDB4E10 and, to a less extent, the MAB2166-2B7 assay detection was found to be poly-Q independent. Finally, the most surprising result was that all assays using the MW1 antibody, both as capture and detection, lost their selectivity for mHTT with the notable exception of the MW1-MAB2166 assay in which a clear separation between the JHD/HD and normal samples was present. The loss of selectivity did not result in overlapping dilution curves among the six fibroblasts, like for the poly-Q independent assays, but rather subject-specific separation.

**Figure 6:**
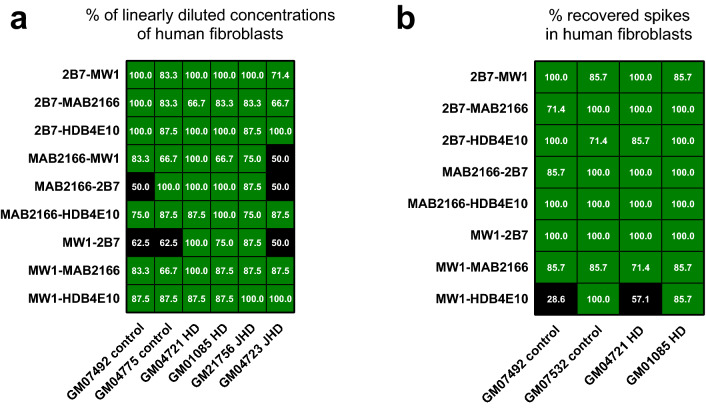
Dilution linearity and spike recovery in wild type, HD and JHD human fibroblast lysates. Black cells are antibody pairs that did not meet the 67% acceptance criteria (see text), green cells show antibody pairs with achieved dilution linearity and/or spike recovery. (**a**) Two control GM04775 (24/17) and GM07492 (21/18), two HD GM04721 (49/36) and GM01085 (45/23) and two JHD GM04723 (70/20) and GM21756 (66/18) fibroblast cell lysate were diluted for 7 dilution points and assayed via SMC using the antibody pair reported on the x-axis. The percentage of the dilution points within the ± 20% RE% is reported in each cell of the heat-map. The acceptance threshold is 66.7%; all conditions passing this threshold are coloured in green. All data for each matrix and each assay are reported in the supplementary figure S-7, calculations were carried out as reported for Fig. 4. (**b**) Two controls GM07492 (24/17) and GM07532 (23/19), two HD (GM0185 (45/23) and GM04721 (49/36) fibroblast lysate were spiked with a serial dilution of the recombinant FL HTT Q46 and assayed via SMC using the antibody pair reported on the x-axis. The percentage of the recovered spikes, within one matrix, is reported in each cell of the heat-map. The acceptance threshold is 66.7%; all conditions passing this threshold are coloured in green. All data for each matrix and assay are reported in the supplementary figure S-8.

To better characterize the validity of the mHTT/HTT detection in human HD fibroblasts the assays with acceptable sensitivity were subjected to the dilution linearity and spike recovery tests. Almost all assay signals were linearly diluted with the exception of the MAB2166-MW1, the MAB2166-2B7 and the MW1-2B7 assays for which the dilution linearity test did not pass for all tested samples (S-7 and Fig. 6a). With regard to spike recovery, JHD fibroblasts were excluded as matrix due to their high endogenous mHTT signal. FL Q46 HTT was spiked as reference analyte. All assays that were linearly diluted passed the spike recovery test, both in normal and HD fibroblast lysates. The only exception being the MW1-HDB4E10 assay which was found to recover around 80% of spikes at most dilutions (S-8 and Fig. 6b).

**Figure 7:**
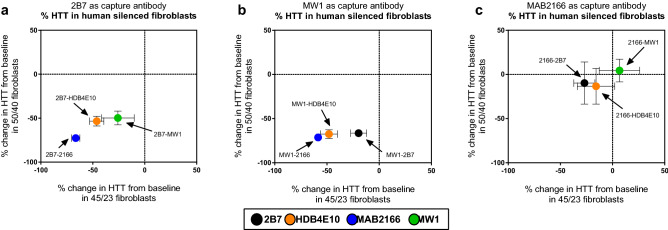
Assay specificity for HTT/mHTT in cells. The percentage signal decrease of HTT silenced samples with respect to control treated samples was measured via SMC by multiple antibody combinations and is reported on the x-axis for GM01085 (45/23) and on the y-axis for GM04857 (50/40). All numeric results are available in S-9. The leftmost group reports the result of the assays with the 2B7 antibody as capture. The middle panel reports the result of the assays with the MW1 antibody as capture, whereas the rightmost panel reports the result of the assays with the MAB2166 as capture antibody. Each colour represents a different detection antibody as reported in the legend. Each result is presented as the average and standard deviation of three independent experiments. Validation of the silencing via Western blot is presented in the supplementary material S-9. Each experimental point is reported as the average and standard deviations of a triplicate.

Further to the above tests, the specificity of these assays for mHTT/HTT was investigated by lowering fibroblast endogenous mHTT/HTT by HTT-directed siRNA. To this aim two subject fibroblast cells were chosen: The GM01085 (45/23) and the GM04857 (50/40). The latter, compound heterozygous HD line, was chosen in order to best determine the selectivity for the readout for mHTT. mHTT/HTT lowering by siRNA was found to be effective at protein (at the FL molecular weight) level for both cells (S-9). Nonetheless, while the staining of the MAB2166 was clean all other antibodies showed numerous bands at lower molecular weights most of which were not affected by the lowering treatment. In particular, the 4C9 antibody produced an extremely intense unspecific band at around 60 kDa. We do not expect these bands to influence the readout. In fact, the detection specificity is dependent on the analyte recognition both by the capture and detection antibodies and the chance that unspecific bands overlap is extremely low. The SMC assay specificity results are summarized in Fig. 7 where the degree of mHTT/HTT silencing, with respect to the scramble treated control, is reported for GM01085 (45/23) on the x-axis and for GM04857 (50/40) on the y-axis. Each graph represents one capture antibody (i.e. 2B7, MW1, and MAB2166) whereas the colour of each dot represents the detection antibody. The obtained results can be summarized as follows: (i) the assays with the MAB2166 as detection are the most selective for mHTT/HTT (blue dots) followed by the one with the HDB4E10 as detection (orange dots) (ii) Surprisingly, assays with the MAB2166 as capture antibody (right panel) are not specific for mHTT/HTT lowering (iii) the 2B7-MW1 and the MW1-2B7 seems to be more sensitive in detecting mHTT lowering in the compound heterozygous (GM04857) cells than the heterozygous one (GM01085) suggesting a certain specificity for mHTT.

### BACHD rat life-span measurement of mHTT/HTT levels in brain and CSF samples

**Figure 8:**
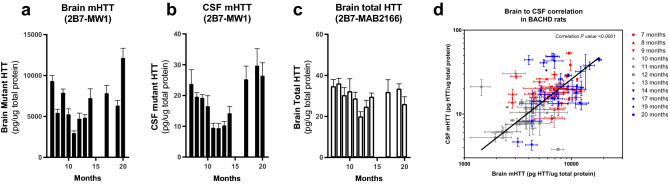
CSF and brain HTT and mHTT levels in a BACHD life-span study. (**a**) Brain mHTT levels as measured by the 2B7-MW1 SMC assay in a cohort of c. 10 BACHD rats for time point. (**b**) CSF mHTT levels as measured by the 2B7-MW1 SMC assay in a cohort of c. 10 BACHD rats for time point. (**c**) Brain levels of total HTT as measured  by the 2B7-MAB2166 SMC assay in a cohort of c. 10 rats for time point. (**d**) Correlation (non-parametric Spearman correlation, two-tailed *p* value, confidence interval = 95%, *p* < 0.0001, r^2^ = 0.52) of mHTT levels between brain (x-axis) and CSF (y-axis) at multiple time points (symbol shapes) and age periods (colours) as measured by the 2B7-MW1 SMC assay. Every experimental point is reported as average and standard deviation of a technical triplicate. All numeric values are available in the supplementary material S-10.

The 2B7-MW1 antibody pair, which is widely used to measure mHTT^16,19,20^, was confirmed in the present work to be specific for mHTT and compliant with the dilution linearity and spike recovery requirements. In addition, the 2B7-MAB2166 was shown to be most specific poly-Q and HTT length independent assay compliant with the validation criteria of the present work. As a consequence, they were chosen to measure mHTT levels in the brain of BACHD rats in a life-span experiment. BACHD rats were used for this study due to the relatively larger amount of CSF in these animals when compared to HD mouse models. The aim of the present experiment is to demonstrate the correlation between mHTT in brain and CSF, The nature of the transgenic model makes the measured mHTT/HTT trends not directly translatable to the human disease. mHTT levels were tested in the CSF of the same animals as well by the 2B7-MW1 assay in order to possibly establish a brain to CSF correlation. The study started at seven months of age as this time was considered the first one at which a sufficient amount of CSF could be collected for detection. Animals were sacrificed at different time points (months) in order to have ten animals per group. For each animal brain a piece of the left hemisphere frontal cortex was sampled and homogenized to be tested for mHTT and total HTT concentration. The CSF samples were clarified and then diluted for testing. The total protein content of both brain and CSF was used to normalize the obtained results. mHTT levels in the brain and CSF of BACHD rats were found to initially decrease during the early life stage and to increase later during the animal life (Fig. 8a,b). On the same set of brains, the 2B7-MAB2166 assay was used to investigate total HTT levels variation during time. Differently from what was observed for mHTT, total HTT levels do not show strong variations over time, though an underlying trend similar to the mHTT one could be observed (Fig. 8c). Finally, statistically significant correlation between brain and CSF mHTT levels was established (*p* < 0.0001, r^2^ = 0.52, Fig. 8d). Unfortunately, due to the limited sample volumes that can be obtained, no CSF was available for the assessment of total HTT in rat CSF after mHTT levels detection, therefore the same correlation was not studied.

### Time course mHTT/HTT detection in multiple tissues of wild type and Q175 mice

**Figure 9:**
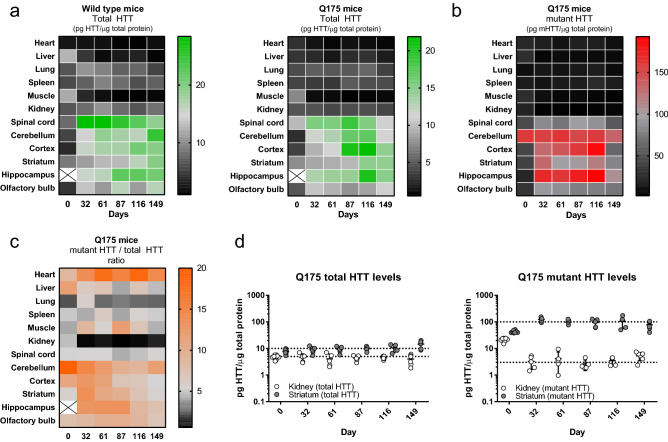
Time-course HTT and mHTT levels in wild type and Q175 tissues. (**a**) Total HTT levels, as measured by the 2B7-MAB2166 SMC assay in different tissues and at different time points, are reported as heat maps for wild type (left) and Q175 KI (right) mice. Each colour represents the average levels of four to six animals. (**b**) mHTT levels, as measured by the 2B7-MW1 SMC assay in different tissues and at different time points, are reported as heat map for Q175 KI mice. Each colour is the average levels of four to six animals. (**c**) The ratio between mHTT and total HTT in Q175 samples is reported for each analysed tissue and time point as heat map. (**d**) Total HTT (left panel) and mHTT (right panel) levels for each animal are presented for the striatum (grey) and kidney (white) of Q175 animals. All numeric values are available in the supplementary material S-11.

Multiple tissues (Fig. 9) were sampled from wild type and Q175 KI mice and subjected to mHTT (2B7-MW1) and total HTT (2B7-MAB2166) detection. Each tissue was taken every month starting from newborn animals up to the fifth month of life for a total of six time points per tissue. Four to six animals (both wild type and HD) were sacrificed at each time point. Total HTT detection on both wild type and Q175 mice showed that, as expected, CNS related tissues have a high expression of HTT with respect to non-CNS tissues with heart and muscle being the lowest HTT expressing tissues analyzed (Fig. 9a). With respect to time, total HTT measurements resulted in slightly different levels between wild type and Q175 animals. In fact, while total HTT levels are generally increasing during aging in CNS wild type tissues (with the exception of the spinal cord), they seem to peak at the third-forth month of life and then decrease for Q175 mice (Fig. 9a). This was also observed by others in a longer age series of Q175 mice where mHTT levels significantly decreased at the 9 and 14 month ages in whole brain lysates^35^. This trend observed for mHTT in Q175 mice suggests that the mHTT contribution could be generating the total HTT trend reported above. Finally, the mHTT/total HTT ratio was calculated for Q175 KI mice (Fig. 9b). As expected, due to mHTT assay artefacts described in the present work, mHTT/total HTT levels are always greater than one. Although this is a paradox, it has to be considered that the mHTT quantification is a relative quantitative measurement and should not be taken as an absolute value. Having clarified this aspect, a clear decrease in mHTT/total HTT levels with time is detected for CNS tissues (with the exception of the spinal cord) supporting the above concept that mHTT soluble levels are decreasing faster than total HTT. Surprisingly, kidney level trends stand out of all other tissues. In fact, although total HTT is expressed in kidney to a level which is marginally lower than striatum (Fig. 9c), its mHTT levels are c. 30-fold lower (Fig. 9d) suggesting that there is a tissue specific regulation of mHTT homeostasis in this animal model. All numerical results are available in the supplementary material S-11.

## Discussion

Here we investigated mutant and total HTT levels in tissues of preclinical HD models at different ages to generate knowledge on HTT protein homeostasis in various tissues. To this aim we set out to survey several anti-HTT antibodies to validate the optimal HTT detection assays. During this process we generated several relevant findings on the use and behaviour of HTT-directed immunoassays. As a consequence, we decided to extend the scope of the work to provide guidelines for the use of quantitative mHTT/HTT immunoassays. Table 1 summarizes the findings of the present work including the selectivity testing on mHTT/HTT in silenced primary human fibroblasts. It is important to note that the validity of these findings must be considered with respect to the amount of sample analysed (i.e. < 4 µg). So, as an example, it cannot be excluded that the specificity for mHTT of some of the assays is impaired at a higher sample load. The following reports our recommendations for the use and HTT directed immunoassays.

**Table 1:**
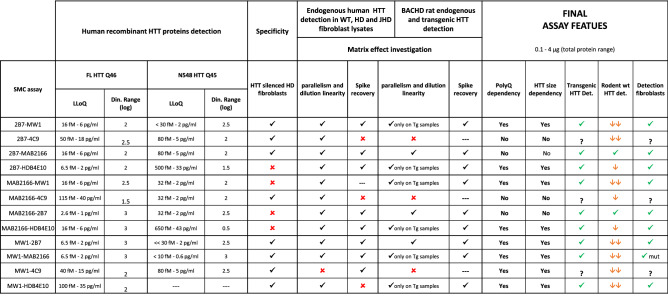
Summary of all tests carried out for each antibody pair (first column).

The symbols in the table are used as follows: “---”: the test was not carried out, green “✔”: the test successfully passed, red “✘”: the test did not passed, “?”: impossible to call, “↓”: low detection, “⇊”: very low detection. Additional abbreviations are: WT (wild type), HD (Huntington’s disease primary samples), JHD (juvenile Huntington’s disease primary samples), Tg (transgenic), mut (mutant HTT).

### Assay performance change between reference proteins and cell and tissue samples

For most of the tested assays, no or only minor differences in the performance between the detection of the reference proteins and the biological samples was observed. We were surprised by the lack of selectivity by the 2B7-MW1 in these cells though the JHD show slightly higher signals than HD and wt. We believe this has to do with the specific form of HTT/mHTT present in these cells. In addition, assays containing the 4C9 antibody are an exception. In fact, a sensitivity drop was observed both in rat brain homogenates (BACHD and wt) and in primary HD fibroblasts with respect to reference proteins. While issues in detecting the 4C9 recognized PRD on the endogenous rodent HTT were expected due to species-specific differences in this domain^28^, it was somewhat surprising that this antibody was not performing well on the human HTT transgene of the BACHD rats or in human HD primary fibroblasts. Given that other reports do not seem to identify the same issue we tend to attribute this to the SMC technology and the relative low amount of sample used in these studies.

### Full length HTT (1-3144) versus HTT (1-573) detection

All assays using the MW1 antibody either as capture or as detection (with the exception of the MW1-HDB4E10 assay) were found to better detect the N573 fragment than the FL proteins. This finding is in line with previous reports^19^ and must be considered to properly interpret MW1 detected mHTT steady state levels in pre-clinical and clinical studies. In fact, cross-subject differences in HTT detection may not be only attributable to different protein levels and poly-Q expansion, but also to potential differences in protein cleavage/clearance. Similarly, in longitudinal studies, changes in the assay signal could be dependent both of expression changes but also HTT cleavage/degradation and somatic expansion. The HDB4E10 antibody was claimed to recognize an epitope between amino acid 1844 and 2131, as a consequence it should have recognized the FL proteins only. Surprisingly, it was found to detect the both the FL and the N573 fragment with better sensitivity for the FL. This seems to suggest that this antibody has ambiguous specificity for both the HTT N-terminal and the claimed C-terminal epitope. Further work is required to elucidate this hypothesis.

### Poly-Q dependent signal increase

All assays employing the MW1 and the HDB4E10 antibodies were found to produce signals which increase with the increase of the poly-Q expansion. While this was expected for the MW1 based on various previous reports^18,19,20,36^, however it was not for the HDB4E10. As per previous paragraph the HDB4E10 specificity will request further investigations which are outside the scope of the present work. The poly-Q dependency of these assays must be carefully considered when comparing results among different subjects bearing different poly-Q as, for example, in human studies or in studies aimed at comparing HTT/mHTT levels among different animal models. In fact, in the absence of a reference protein for every poly-Q expansion, HTT levels back-calculated on a single reference protein will be relative-quantitative rather than absolute. Additionally, selected biosamples may contain a mixture of HTT length species complicating quantitation using a single reference standard protein.

### mHTT/HTT selectivity in primary HD fibroblasts

The selectivity of the present assays for mHTT/HTT was investigated in primary HD and control human fibroblasts by mHTT/HTT knock down. Despite these cells not being a primary disease tissue, they represent a convenient in vitro system expressing an endogenous human HD relevant mHTT protein analyte. Among the reported results, the behaviour of the MAB2166 antibody, when used as capture, was the more unexpected. In fact, while as detector (in pair with 2B7 or MW1) it was found to be the most sensitive antibody for the quantification of mHTT/HTT lowering; as capture it was ineffective paired with all detection antibodies. As an example, the 2B7-MAB2166 is clearly mHTT/HTT selective while the MAB2166-2B7 is not. We tend to exclude this phenomenon to be related to poly-Q dependency of the antibodies. In fact, the disconnect between the MAB2166 used as detection or as capture is true for both the 2B7-MAB2166 Vs MAB2166-2B7 and MW1-MAB2166 Vs MAB2166-MW1. The former being poly-Q independent and the latter being poly-Q dependent. If we were to speculate, we would say this to be related to the MAB2166, when immobilized on magnetic beads, having different affinities for different mHTT/HTT forms with potentially different half-lives resulting in the extraction, with high specificity, of a detectable long-lived form of mHTT/HTT. Though intriguing, the elucidation of this phenomenon is outside the scope of the present work. Meanwhile we do not recommend using MAB2166 as capture antibody in SMC assays.

### Summary of recommendations and the paradox of mHTT levels

The following recommendations should be considered while detecting mHTT/HTT by SMC with the antibodies described in the present work:

- The HDAB4E10 and the 4C9 antibodies must be used with caution as their selectivity, specificity, and sensibility could greatly vary among reference proteins, human cells, and rodent tissues.
- mHTT selective assays using the MW1 antibody give relative-quantitative measurements rather than absolute results due to the poly-Q and protein size dependent signals. This phenomenon leads to the paradox of total HTT levels being apparently lower than mHTT levels.
- The MAB2166 is specific and selective for mHTT/HTT and produces poly-Q and protein size independent results when paired with the 2B7 antibody. Nonetheless, when used as capture it did not result in an appreciable selectivity for mHTT/HTT.
- The 2B7-MW1 and the 2B7-MAB2166 assays are the recommended assays for the detection of mHTT and total HTT respectively.

### Time course of mHTT/HTT levels in Huntington’s disease murine models

BACHD rats were used to determine the mHTT/HTT brain and CSF levels due to the relatively larger amount of CSF in these animals when compared to HD mouse models. The 2B7-MW1 and the 2B7-MAB2166 assays were used to detect mHTT and total HTT respectively in the brain of BACHD rats at multiple time points during their life span. A decrease of mHTT in brain of c. 50%, with respect to 7-month levels was observed at around 10-months of life. This decrease is followed by an increase back to initial levels at 20-months. While it is difficult to speculate on the pathophysiological significance of mHTT steady state level variations, also because of the influence of the aggregation process that is not measured here, it is important to consider them in mHTT/HTT lowering approaches in order to best interpret their results. With regards to the brain-CSF correlation, mHTT levels detected in the CSF were correlated to those in the brain confirming the fact that CSF is a good surrogate tissue for brain. Finally, when total HTT was evaluated in the brain using the 2B7-MAB2166 assay, its variation during the animal life was found to be minimal. This is in line with the notion that these animals have two mouse wild type alleles and one transgenic human mHTT allele whose variation is likely diluted in the former type. This finding suggests that the endogenous wild type HTT and the human transgene mHTT steady state levels may be controlled by different mechanisms.

An investigation of the mHTT/HTT levels in CNS compared to peripheral tissues was carried out in Q175 mice since this model is widely used for in vivo HD studies. For this reason, understanding mHTT/HTT levels, as single measures and as a ratio between the two measures, may provide relevant insights not only as indications of the efficacy of a therapeutic intervention, but also in terms of relevant tissues for the study of mHTT/HTT expression. In this work we show that the ratio between mHTT and total HTT can strongly vary in time as well as between diseased tissues, such as the striatum, and less-affected tissues such as the kidney. These types of observations, which are enabled by the use of the assays presented here, can open the field to investigations of the proteostasis of mHTT/HTT in tissues thus generating new hypothesis for future meaningful intervention.

## Material and methods

### Recombinant proteins

Purified recombinant proteins were produced as previously published. The large fragment HTT protein (amino acid 1-573) with Q23, Q45, or Q73 N573 according to Macdonald, et al^18^ and the full length HTT protein with Q17 and Q46 according to Huang et al^36^.

### Antibodies

The MW1 antibody recognizing the expanded polyQ HTT domain was developed by Dr. Paul Patterson^24^. The 2B7 antibody to the HTT amino terminus and 4C9 antibody to the HTT proline rich domain were generated and characterized as previously described^16^. The MW1, 2B7, and 4C9 antibodies were all obtained through the CHDI Foundation (New York, NY). The MAB2166 antibody was obtained from Millipore (cat. n. MAB2166), and the HDB4E10 antibody was obtained from AbD Serotec (Bio-Rad Laboratories; cat. n. MCA2050). The 2B7, MW1, and MAB2166 antibodies were conjugated to magnetic particles (MPs) using the Millipore MPs conjugation kit to a final concentration of 25 μg/mg of MPs. The 2B7, MW1, 4C9, MAB2166, and HDB4E10 antibodies were conjugated with the detection reagent labelling kit (Millipore) to a final concentration of 1 μg/μl. Conjugated/labeled antibodies were diluted in Sample buffer (Candor, 105 500), prior to performing the assay.

### Cells and tissues

HD and non-HD fibroblasts were obtained from The Coriell Institute for Medical Research (Camden, NJ, USA) and cultured in MEM (GIBCO, 21090) added with 15% fetal bovine serum and 1% PenStrep (Gibco, 15140122). Table S-2 in the supplementary material provides the details of the cells used in this study. HTT lowering was carried out using the HTT-specific siRNA from SIGMA (cat. num. SASI_HS01_00241076) with Lipofectamine 2000 (Invitrogen) as transfecting reagent. Transfected cells were collected 48 h after treatment and lysed. Lysis was performed in PBS, 0.4% Triton-X and Protease Inhibitor cocktail tablets (complete from Roche). Brain tissues from the BACHD rat model^21^ and wild type controls were obtained from Charles River Laboratories International, Inc. For homogenization, the forebrains were diluted in lysis buffer as per above and transferred ceramic beads containing vials (Lysing matrix D, MP Biomedicals, cat. n. 116913050). After homogenization with a FastPrep96 homogenizer (MP, USA) by 3 × 30 s cycles at 1600 rpm, samples were stored at − 80 °C overnight and clarified by centrifugation before total protein quantification.

### Animal models and genotyping

zQ175 knockin mice^22^ (Q175) were bred at IRBM and tissue samples were collected in full compliance with the EU Directive 63/2010 and its Italian transposition as well as with all applicable Italian legislation and guidelines. IRBM is authorized by the Italian Ministry of Health and local veterinary authority to house, breed, and use laboratory rodents for scientific purposes. All protocols used at IRBM are approved by the Institutional Animal Care and Use Committee (IACUC). The identification of wild type versus knocked-in animals was carried out via genotyping according to the following procedure. Genomic DNA isolation was performed by isopropanol precipitation from lysates of mice ear punches. Lysis buffer composition: 100 mM Tris pH 8, EDTA 5 mM, SDS 0.2%, NaCl 200 mM, 0.8 mg/mL proteinase K (ThermoFisher Scientific, 25530049). After precipitation, the genomic DNA was resuspended in ultrapure DNase/RNase-free distilled water (ThermoFisher Scientific, 10977035). Approximately 100 ng of genomic DNA were used for the PCR amplification of the knocked-in gene, using Neo1 (5′-gatcggccattgaacaag-3′) and Neo2 (5′-agagcagccgattgtctg-3′) as primers; whereas Hdh1 (5′-cattcattgccttgctgc-3′) and Hdh2 (5′-ctgaaacgacttgagcga-3′) were used for the amplification of the mouse *Hdh* gene. The PCR was carried out using the *Taq* DNA polymerase (ThermoFisher Scientific, 10342) following the manufacturer's instructions, with an annealing temperature of 57 °C. Amplification products were run on 2% agarose gels and revealed by SYBR Safe staining (ThermoFisher Scientific, cat. n. S33102).

Tissue homogenization for HTT protein analysis was carried out as above.

### Immunoassays

The immunoassays were performed as previously described^19^. Briefly, the assay starts with a capture step, where conjugated magnetic particles (MPs) are incubated with samples for one hour, in an Axygen polypropylene V-bottom 96 well plate, previously coated with D2X solution composed of 750 mM NaCl, 6% BSA, 0.8% Triton-X and protease inhibitor cocktail (Complete tablets from Roche). Washes were performed on a magnetic rack, using an Elx washer (from Biotek), in 1 × Erenna Wash Buffer (from Millipore). Afterwards MPs were incubated with the detection antibody (fluorophore conjugated) for one hour. Washes were performed again on a magnetic rack, in an Elx washer, in 1X Erenna Wash Buffer. The MPs were then transferred into a new Axygen 96 well plate to eliminate nonspecific binding to the plastic. After buffer aspiration, the elution buffer (acidic glycine solution, 0.1 M, pH 2.7) was added to the plate for five minutes of incubation under shaking. The eluted detection antibody was transferred to a Nunc 384-well analysis plate and neutralized with neutralization buffer (Tris, 1 M, pH 9). The plate was spun down, sealed and subsequently analyzed with the SMC Erenna Immunoassay System (Millipore).

### Data analysis

Data analysis was performed using GraphPad Prism software in order to obtain standard curve fitting and back-calculations on fitting models. The standard curve was obtained without associating any weight to each standard concentration.

## Supplementary Information

Supplementary Information.
