# Supplementary material for: Analysis of mutant and total huntingtin expression in Huntington’s disease murine models
Source: Sci Rep. 2020 Dec 17;10:22137. doi: 10.1038/s41598-020-78790-5 (PMC7746729; doi:10.1038/s41598-020-78790-5)
Supplement: Supplementary file 1 — Supplementary Information. [file 41598_2020_78790_MOESM1_ESM.pdf]

Supplemental Information

Title

Analysis of mutant and total huntingtin expression in Huntington's disease murine models.

Authors

Fodale Valentina[1‡], Roberta Pintauro[1‡], Daldin Manuel[1], Altobelli Roberta[1], Spiezia Maria Carolina[1], Bisbocci Monica[1], Macdonald Douglas[2*], Bresciani Alberto[1]

Affiliations

[1] Department of Translational Biology, IRBM S.p.A., via Pontina Km 30,600 Pomezia (Roma) – ITALY

[2] CHDI Management/CHDI Foundation, Suite 700, 6080 Centre Drive, Los Angeles (CA) – USA

[‡] These authors equally contributed to this work

***Corresponding author**

Douglas Macdonald (douglas.macdonald@chdifoundation.org) CHDI Management/CHDI Foundation, Suite 700, 6080 Centre Drive, Los Angeles (CA) – USA

S-1 Full-length Western blot of HTT recombinant proteins

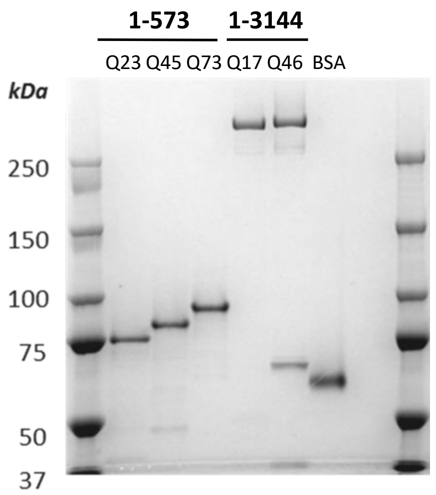

A

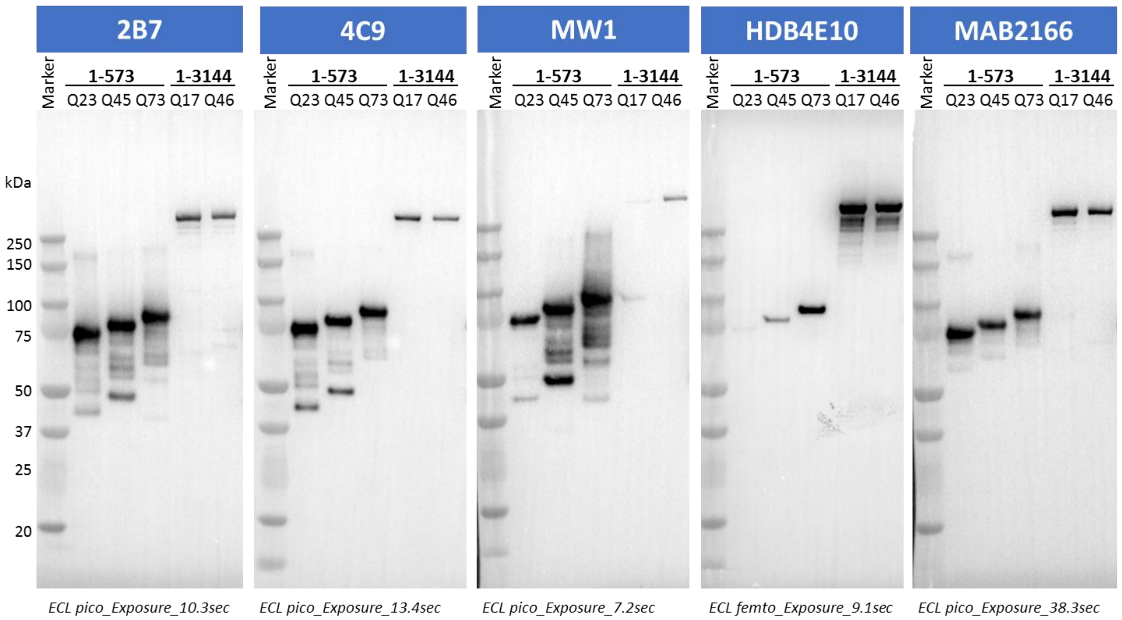

B

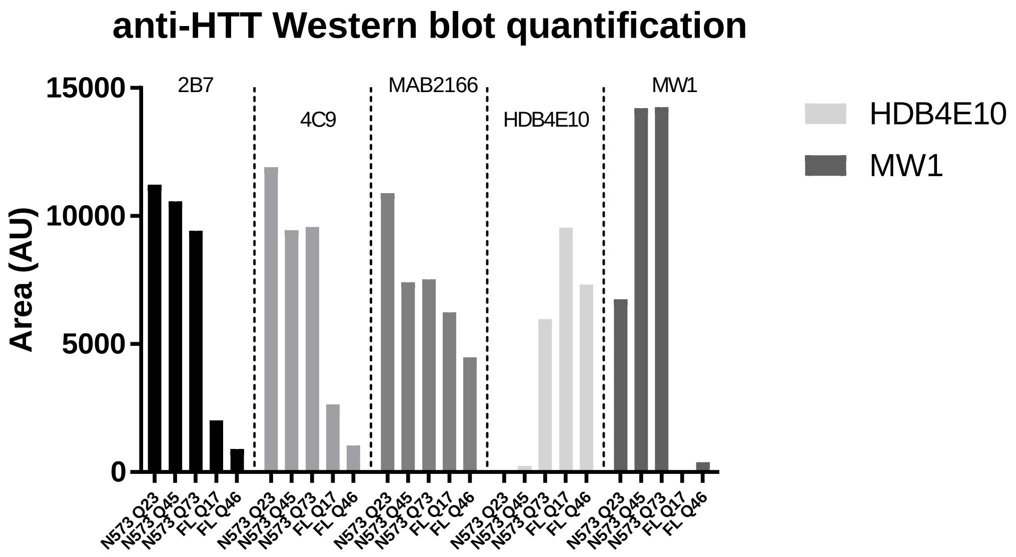

C

(A) Coomassie staining of the five standard protein used in the present work (left to right for each panel: N573 Q22, N573 Q45, N573 Q72, FL Q17, FL Q46, BSA 1 ug). (B) high exposure Western blots of the five standard proteins (left to right for each panel: N573 Q22, N573 Q45, N573 Q72, FL Q17, FL Q46) stained with the five antibodies used in the present work. (C) densitometry of the Western blot HTT bands in panel (B)

S-2 Fibroblast cell information

Catalog_ID	Age of onset	HD	CAG repeats Laragen	Age	Gender	Notes
GM21756	unknown	JHD	66/18		Female	Clinically affected;
GM04723	14	JHD	70/20	19	Female	clinically affected; neuro exam 3/82 shows bradykinesis, juvenile features of HD with markedly reduced saccadic velocity, Parkinsonian features, and marginal dystonia;
GM07492	unknown	normal	21/18	17	Male	
GM07532	unknown	normal	23/19	16	Female	Caucasian mother and Japanese father;
GM04857	unknown		50/40	23	Female	clinically affected; onset at age 28 years; 9 affected sibs and 2 with probable HD; neurological exam 3/82 shows bradykinesia;
GM04721	35	HD	49/36	37	Female	clinically affected; neuro exam 3/82 shows chorea & facial twitches; 1st cousin to proband;
GM01085	35	HD	45/23	44	Male	moderate ataxia, chorea, dysarthria, and dysphagia;
GM04693	41	HD	47/23	33	Male	clinically affected;
GM04775	unknown	normal	24/17	46	Female	

S-3 Performance of HDB4E10 and 4C9 containing assays against rat brain homogenates

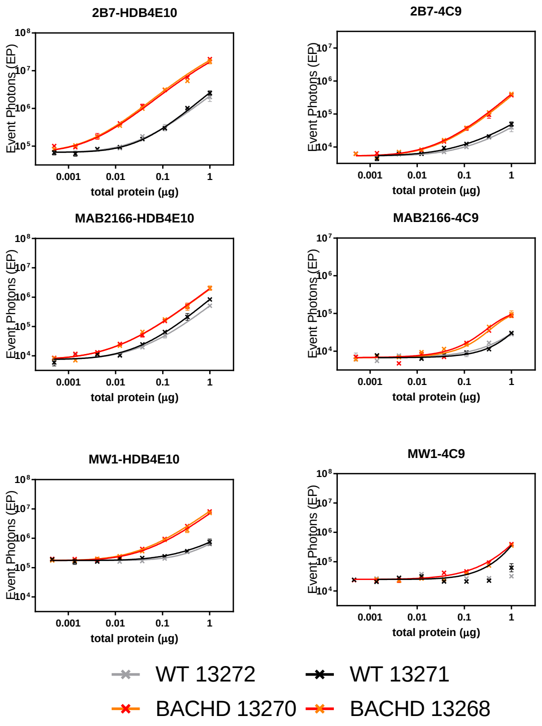

Two wild type rats (13272 and 13271) and two BACHD rats (13270 and 103266) brain homogenates were diluted starting 1 from 6.7 µg/ml of total protein for 7 dilution points. Each dilution curve was tested via SMC using the antibody pair reported in the title of the graph.

S-4 Dilution linearity in wild type and BACHD rat brain homogenates

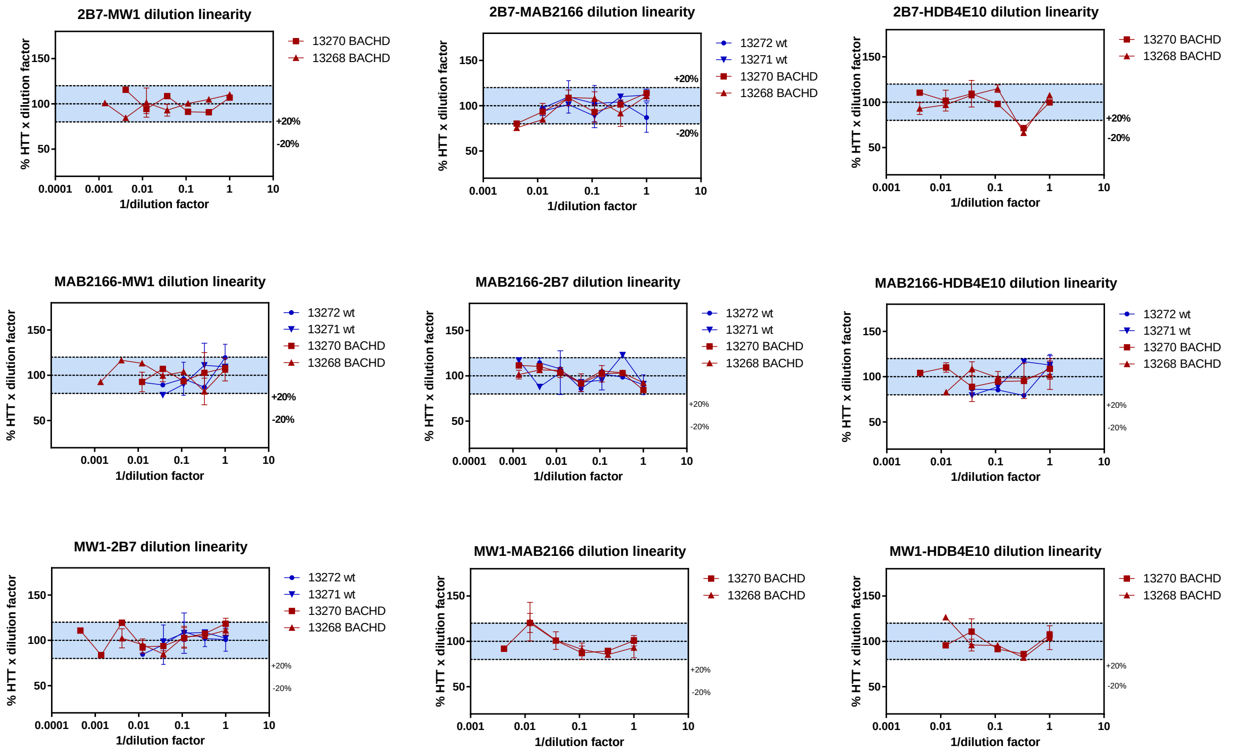

Two wild type rats (13272 and 13271) and two BACHD rats (13270 and 103266) brain homogenates were diluted starting 1.3 µg/ml of total protein for the 2B7-MW1 assay and from 6.7 µg/ml of total protein for all other antibody pairs for 7 dilution points. Each dilution curve was tested via SMC using the antibody pair reported in the title of the graph. The graphs show the percentage recovery (x-axis) with respect to the average concentration of each dilution multiplied for the dilution factor (y-axis). The light blue band represent the acceptable ± 20% RE% range. Each point is represented as the average and standard deviation of three independent replicates.

The following examples show the process of determining the dilution linearity of the 2B7-MW1 for the 13270 BACHD brain.

Conc (pg mHTT/ug total protein)	Dilution factor		Conc. X Dil. factor	RE% w.r.t. average
5717.27	1.00	==>	5717.27	5.61
1617.40	3.00	==>	4852.20	-10.37
542.56	9.00	==>	4883.05	-9.80
215.05	27.00	==>	5806.33	7.26
62.25	81.00	==>	5041.90	-6.86
25.43	243.01	==>	6180.03	14.16
		Average	**5413.46**	

S-5 Spike recovery in wild type and BACHD rat brain homogenates.

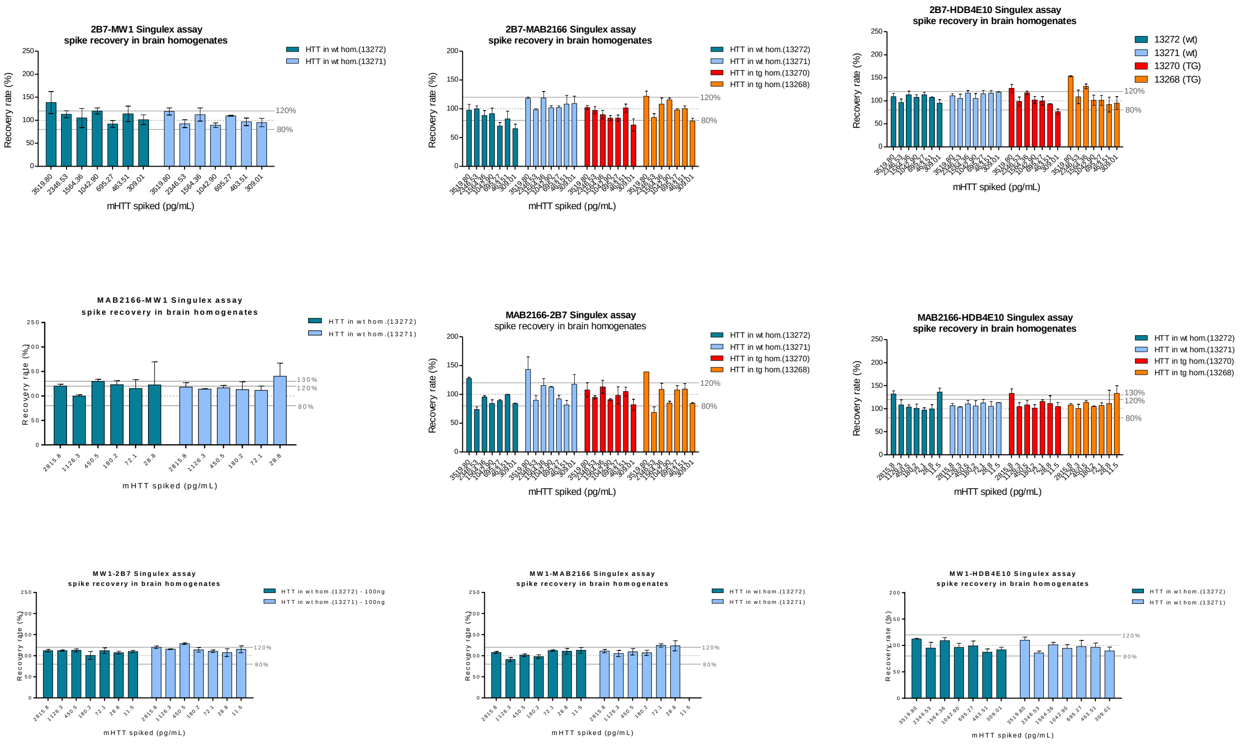

Two wild type (13272 and 13271) and two BACHD (13270 and 103266) brain samples were spiked with a serial dilution of the recombinant FL HTT Q46 and assayed via SMC using the antibody pairs reported in the title of each graph. The amount of brain homogenate used for each assay is reported in the table below. The percentage of the recovered spikes for each spike dilution is reported in the x-axis, while the nominal spike concentration is reported on the y-axis. Each point is represented as the average and standard deviation of three independent replicates.

	Starting total protein concentration	
assays	ug/mL wt	ug/mL HD
2B7-MW1	0.13	X
2B7-MAB2166	0.33	0.33
2B7-HDB4E10	0.67	0.67
MAB2166-MW1	0.67	0.20
MAB2166-2B7	0.33	0.33
MAB2166-HDB4E10	0.67	0.67
MW1-2B7	0.67	0.67
MW1-MAB2166	0.67	0.67
MW1-HDB4E10	0.67	0.20

S-6 Performance of HDB4E10 and 4C9 containing assays human fibroblast lysates

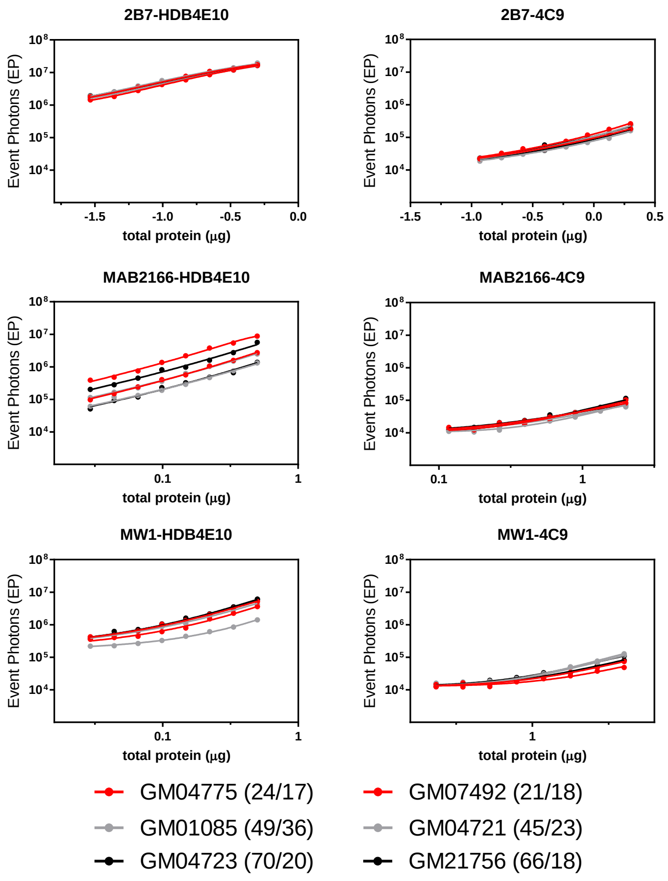

Two control GM04775 (24/17) and GM07492 (21/18), two HD GM04721 (49/36) and GM01085 (45/23) and two JHD GM04723 (70/20) and GM21756 (66/18) fibroblast cell lysates were diluted starting from the total protein concentrations reported in the table in S-7. Each dilution curve was tested via SMC using the antibody pair reported in the title of the graph

S-7 Dilution linearity in HD and JHD human fibroblast lysates

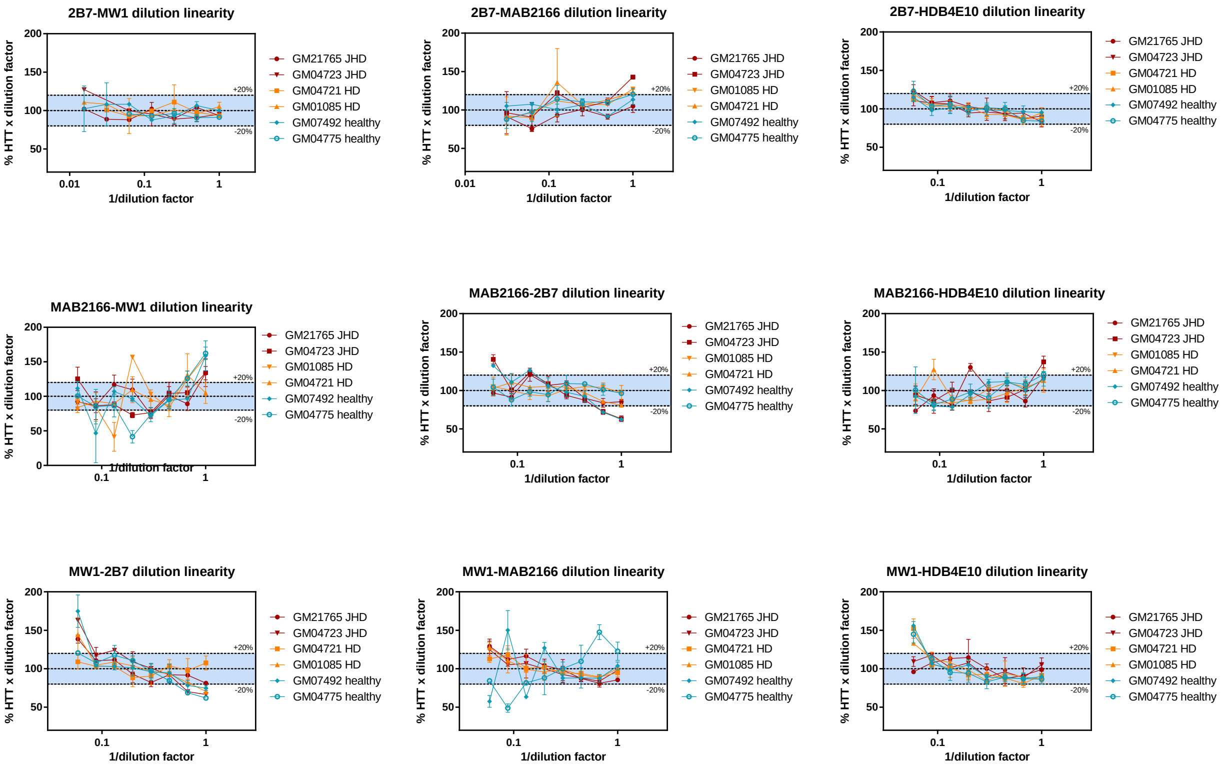

Two control GM04775 (24/17) and GM07492 (21/18), two HD GM04721 (49/36) and GM01085 (45/23) and two JHD GM04723 (70/20) and GM21756 (66/18) fibroblast cell lysates were diluted starting from the total protein concentrations reported in the table below. Each dilution curve was tested via SMC using the antibody pair reported in the title of the graph. The graphs show the percentage recovery (x-axis) with respect to the average concentration of each dilution multiplied for the dilution factor (y-axis). The light blue band represent the acceptable ± 20% RE% range. Each point is represented as the average and standard deviation of three independent replicates.

	Starting total protein concentration
assays	ug/mL
2B7-MW1	3.33
2B7-MAB2166	13.33
2B7-HDB4E10	3.33
MAB2166-MW1	13.33
MAB2166-2B7	1.67
MAB2166-HDB4E10	3.33
MW1-2B7	3.33
MW1-MAB2166	26.67
MW1-HDB4E10	3.33

S-8 Spike recovery in wild type, HD and JHD human fibroblast lysates

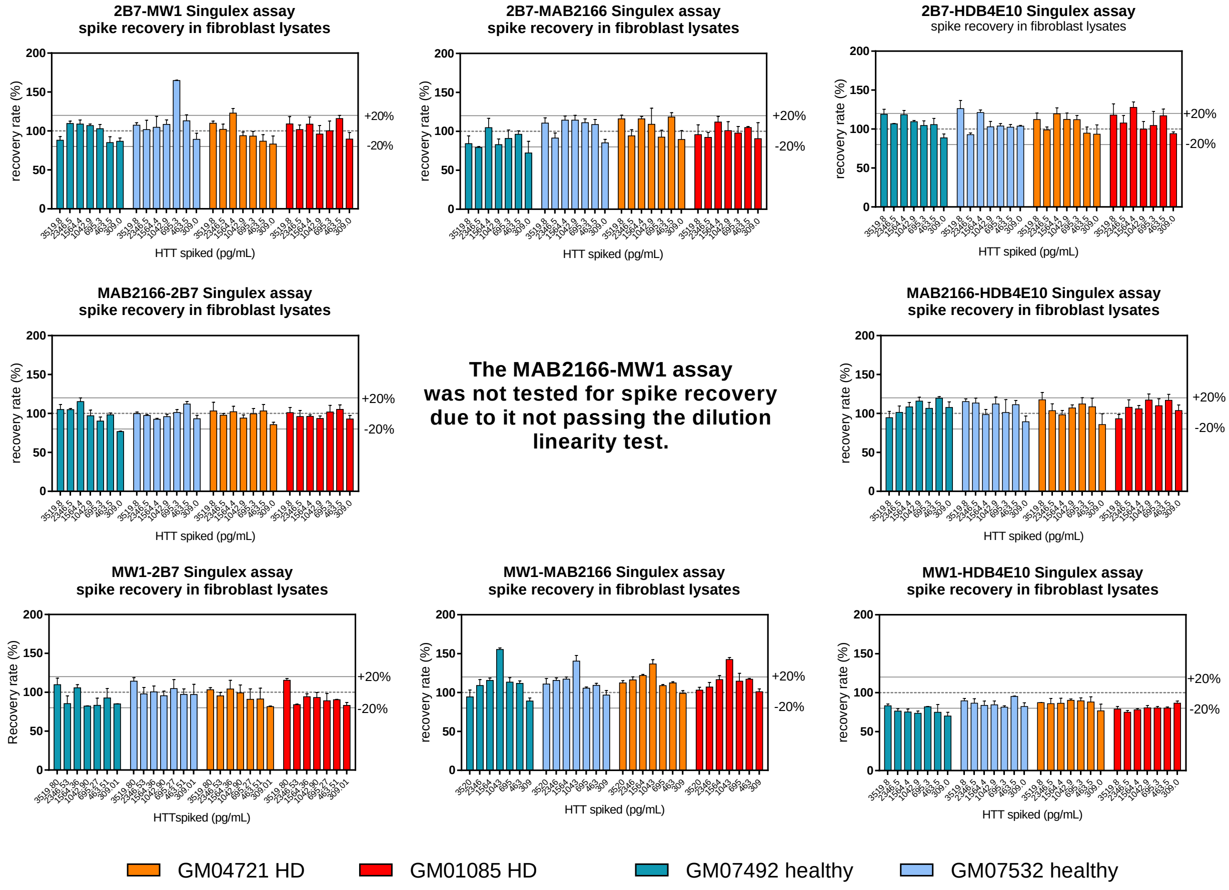

Two control GM04775 (24/17) and GM07492 (21/18), two HD GM04721 (49/36) and GM01085 (45/23) and two JHD GM04723 (70/20) and GM21756 (66/18) fibroblast cell lysates were spiked with a serial dilution of the recombinant FL HTT Q46 and assayed via SMC using the antibody pairs reported in the title of each graph. The amount of brain homogenate used for each assay is reported in the table below. The percentage of the recovered spikes for each spike dilution is reported in the x-axis, while the nominal spike concentration is reported on the y-axis. Each point is represented as the average and standard deviation of three independent replicates.

	Starting total protein concentration	
assays	ug/mL wt	ug/mL HD
2B7-MW1	0.13	0.13
2B7-MAB2166	1.67	1.67
2B7-HDB4E10	0.07	0.07
MAB2166-MW1	X	X
MAB2166-2B7	0.02	0.02
MAB2166-HDB4E10	0.20	0.20
MW1-2B7	0.07	0.07
MW1-MAB2166	8.00	0.67
MW1-HDB4E10	0.07	0.07

S-9 HTT silencing in human fibroblasts

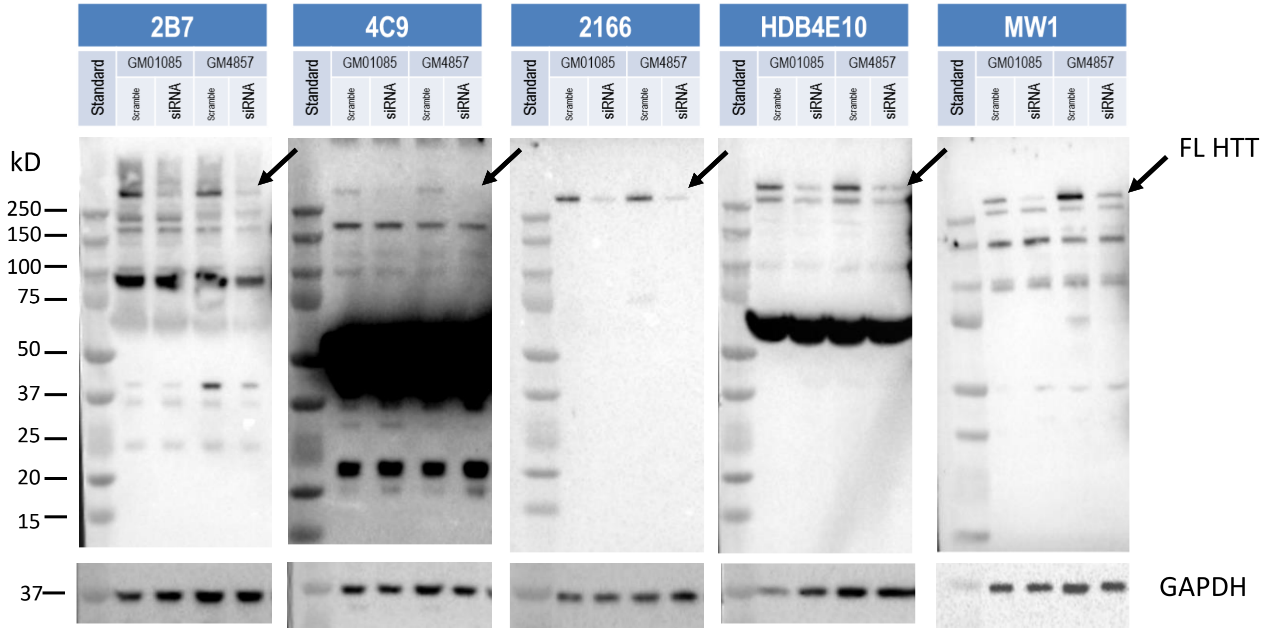

Two fibroblasts cell lines GM01085 (45/23) and GM04857 (50/40) were subjected to HTT silencing via siRNA. Cell lysates form both donors were used to determine the silencing efficiency via western blot suing the antibodies under investigation. The present Western blot does not have a sufficient resolution to allow the detection of the mHTT band at higher molecular weight than the wild type one. The following table reports all the numeric % decrease of HTT detected via SMC with respect to the scramble treated cells.

	GM01085 (45/23)		GM04857 (50/40)	
Antibody pair	% decrease w.r.t scramble	StDev	% decrease w.r.t scramble	StDev
2B7-2166	-66.43	3.93	-72.71	3.43
2B7-MW1	-25.8557	15.76097	-49.8191	7.831169
2B7-HDB4E10	-46.2	6.8	-53.4	5.5
2166-2B7	-26.96	10.1	-9.74	23.87
2166-HDB4E10	-15.7868	17.9155	-13.3887	20.17093
2166-MW1	6.7	19.46	4.45	12.87
MW1-2166	-58.47	1.56	-71.31	2.17
MW1-2B7	-19.68	7.6	-66.45	2.53
MW1-HDB4E10	-48.09	8.11	-67.53	4.87

S-10 CSF and brain HTT and mHTT levels in a BACHD life-span study

	BRAIN mHTT (2B7-MW1)			CSF mHTT (2B7-MW1)			BRAIN total HTT (2B7-MAB2166)		
Month	Avg	SD	N	Avg	SD	N	Avg	SD	N
7	9296.72	2932.29	18	23.65	14.20	9	34.75	14.57	20
8	5408.79	2011.28	19	19.54	5.50	15	36.08	10.64	19
9	7876.58	1521.02	10	19.17	3.54	10	30.39	11.61	10
10	5224.18	2107.52	9	16.47	8.46	6	32.29	13.63	5
11	2930.50	959.90	10	9.47	4.87	10	28.73	9.75	10
12	4690.37	1761.62	6	9.35	4.04	6	20.07	6.37	8
13	4812.71	1216.79	9	10.21	4.10	9	24.74	10.39	11
14	7213.60	3292.98	8	14.16	6.52	8	29.58	5.46	9
17	7809.51	3141.59	10	25.20	13.13	9	31.97	14.58	6
19	6305.68	2094.90	10	29.64	15.82	8	33.53	7.82	11
20	12127.24	4098.56	11	26.35	11.28	7	26.06	12.32	12

Numerical values of the CSF and brain HTT and mHTT levels in a BACHD life-span study

S-11 Time-course HTT and mHTT levels in wild type and Q175 tissues

Total HTT wt pg HTT/ug total protein)

Days	0			32			61			87			116			149		
	Avg	SD	N	Avg	SD	N	Avg	SD	N	Avg	SD	N	Avg	SD	N	Avg	SD	N
Heart	2.428	0.549	8	2.191	0.801	7	2.004	0.665	7	1257	0.545	6	1010	0.393	7	1893	0.448	6
Liver	12.580	3.601	5	4.473	2.169	6	1710	1631	5	2.642	1025	6	3.194	0.708	5	1237	0.484	3
Lung	6.383	0.945	7	10.473	1297	6	9.408	1266	5	7.240	1044	6	7.637	1789	6	5.370	0.892	5
Spleen	5.770	2.045	8	7.242	1035	5	9.325	1364	6	8.032	2.023	5	6.100	3.466	6	7.210	1408	4
Muscle	11984	2.678	7	3.585	0.519	4	1951	1601	7	1005	0.992	6	1240	1123	6	1512	0.596	5
Kidney	6.550	1136	6	8.008	1366	6	10.412	2.261	5	7.890	1674	7	6.482	2.195	5	8.282	0.952	6
Spinal cord	7.881	3.848	7	28.422	5.308	6	28.363	5.351	3	25.368	1965	4	22.510	5.338	3	18.953	3.318	4
Cerebellum	5.343	1224	8	14.460	5.344	5	18.980	6.725	6	17.592	5.055	6	16.297	4.730	7	25.048	5.366	4
Cortex	3.659	0.353	8	15.588	3.729	6	17.537	2.443	6	17.593	3.111	6	20.923	2.885	6	19.565	2.836	4
Striatum	8.889	1253	8	11.185	2.270	6	12.916	3.113	5	12.924	2.157	7	16.315	4.864	6	19.702	2.664	5
Hippocampus				15.315	1351	2	16.387	3.202	3	22.273	3.260	3	22.220	3.528	3	20.923	1839	3
Olfactory bulb	4.373	0.524	7	13.584	5.893	7	10.875	4.258	6	16.512	4.482	6	11.145	2.015	6	16.618	6.487	5

Total HTT Q175 pg HTT/ug total protein)

Days	0			32			61			87			116			149		
	Avg	SD	N	Avg	SD	N	Avg	SD	N	Avg	SD	N	Avg	SD	N	Avg	SD	N
Heart	2.963	0.292	8	1456	1023	5	0.778	0.611	5	1330	0.478	5	1060	0.271	5	0.782	0.299	6
Liver	3.125	1590	8	2.580	1005	5	1586	0.362	5	2.698	2.132	5	2.996	1176	5	1960	0.929	6
Lung	4.326	1342	8	5.198	3.103	5	4.868	1384	5	4.904	1189	5	5.624	1177	5	3.755	2.335	6
Spleen	3.925	1458	8	3.466	0.835	5	3.228	1280	5	5.000	1892	5	4.418	1650	5	4.312	1092	6
Muscle	7.340	3.676	8	1126	1510	5	1030	1263	5	0.502	0.502	5	0.750	0.900	5	0.920	0.510	6
Kidney	4.740	0.884	8	4.624	1679	5	4.058	2.153	5	4.336	1053	5	4.446	0.672	5	3.990	1724	6
Spinal cord	9.790	4.309	8	15.458	3.367	5	15.445	3.571	2	18.742	7.669	5	15.352	5.200	5	10.955	3.490	6
Cerebellum	2.785	0.943	8	11480	5.099	5	13.030	5.293	5	16.680	3.590	4	16.947	5.519	3	11230	5.787	4
Cortex	3.700	0.516	8	9.466	1616	5	11370	1047	5	20.670	5.211	5	21884	8.136	5	14.487	3.478	6
Striatum	7.600	1539	8	8.892	2.691	5	9.266	2.491	5	9.700	1818	5	14.662	9.695	5	14.480	4.522	6
Hippocampus				13.120	2.414	3	13.967	3.961	3	14.287	0.526	3	20.423	0.465	3	14.853	2.857	3
Olfactory bulb	4.873	1662	8	10.358	7.085	5	9.442	4.249	5	7.856	2.945	5	8.644	0.691	5	10.912	5.004	6

mHTT Q175 pg mHTT/ug total protein)

Days	0			32			61			87			116			149		
	Avg	SD	N	Avg	SD	N	Avg	SD	N	Avg	SD	N	Avg	SD	N	Avg	SD	N
Heart	26.048	2.328	8	20.400	1148	5	14.156	2.811	5	20.542	3.418	5	20.078	1842	5	11218	2.254	6
Liver	36.961	23.189	8	16.272	14.954	5	11420	9.397	5	10.924	9.276	5	20.862	18.328	5	8.817	4.225	6
Lung	9.865	3.562	8	29.780	26.698	5	13.522	2.745	5	15.442	6.241	5	15.722	6.643	5	10.097	6.238	6
Spleen	18.574	6.742	8	14.392	9.533	5	20.306	5.041	5	20.162	5.307	5	23.880	7.647	5	20.885	11590	6
Muscle	30.075	8.148	8	9.874	1991	5	5.508	1759	5	6.036	2.198	5	5.256	1962	5	3.923	1800	6
Kidney	21515	3.452	8	3.276	1655	5	4.130	3.186	5	2.592	1116	5	3.492	1095	5	5.255	1807	6
Spinal cord	48.702	8.410	6	78.228	33.194	5	77.440	11484	3	78.320	35.574	5	70.668	34.840	5	56.597	34.600	6
Cerebellum	156.695	13.039	8	170.076	44.537	5	159.700	32.697	5	161694	71964	5	152.414	29.512	5	132.585	24.281	6
Cortex	40.229	5.961	8	122.430	33.961	5	129.180	41413	5	145.114	65.921	5	147.866	79.006	5	72.618	29.507	6
Striatum	42.716	4.722	8	123.700	23.378	5	98.236	21746	5	94.594	31123	5	105.628	50.086	5	77.543	26.573	6
Hippocampus	27.150	15.752	8	170.696	29.983	5	186.664	50.343	5	192.378	68.726	5	177.556	63.563	5	111.213	47.751	6
Olfactory bulb	37.566	12.659	8	88.098	20.344	5	91468	11524	5	78.128	3.397	5	76.292	11552	5	75.685	30.294	6

mHTT/HTT ratio Q175

Days	0		32		61		87		116		149	
	Ratio	SD	Ratio	SD	Ratio	SD	Ratio	SD	Ratio	SD	Ratio	SD
Heart	8.792	0.413	14.011	4.415	18.195	6.592	15.445	2.737	18.942	2.300	14.352	2.534
Liver	11828	3.378	6.307	2.815	7.201	2.750	4.049	2.100	6.963	2.997	4.498	1238
Lung	2.280	0.384	5.729	2.760	2.778	0.434	3.149	0.664	2.796	0.590	2.689	0.962
Spleen	4.732	0.869	4.152	1309	6.291	1316	4.032	0.831	5.405	1189	4.844	1206
Muscle	4.097	0.825	8.769	5.318	5.348	3.031	12.024	5.718	7.008	3.939	4.264	1252
Kidney	4.539	0.395	0.708	0.197	1018	0.426	0.598	0.132	0.785	0.122	1317	0.297
Spinal cord	4.975	0.850	5.061	1079	5.014	0.925	4.179	1143	4.603	1231	5.166	1454
Cerebellum	56.264	6.935	14.815	3.416	12.256	2.493	9.694	2.193	8.994	1862	11806	3.167

Numerical values of the time-course HTT and mHTT levels in wild type and Q175 tissues
